# Wide Biological Role of Hydroxytyrosol: Possible Therapeutic and Preventive Properties in Cardiovascular Diseases

**DOI:** 10.3390/cells9091932

**Published:** 2020-08-21

**Authors:** Chiara D’Angelo, Sara Franceschelli, José Luis Quiles, Lorenza Speranza

**Affiliations:** 1 Department of Medicine and Aging Sciences, University “G. d’Annunzio” Chieti- Pescara, Via dei Vestini 31, 66100 Chieti, Italy; chiara.dangelo@unich.it (C.D.); sara.franceschelli@unich.it (S.F.); 2 Department of Physiology, Institute of Nutrition and Food Technology “José Mataix Verdú” Biomedical Research Center, University of Granada, 18071 Granada, Spain; jlquiles@ugr.es

**Keywords:** hydroxytyrosol, cardiovascular disease, atherosclerosis, oxidative stress, inflammation, Mediterranean diet

## Abstract

The growing incidence of cardiovascular disease (CVD) has promoted investigations of natural molecules that could prevent and treat CVD. Among these, hydroxytyrosol, a polyphenolic compound of olive oil, is well known for its antioxidant, anti-inflammatory, and anti-atherogenic effects. Its strong antioxidant properties are due to the scavenging of radicals and the stimulation of synthesis and activity of antioxidant enzymes (SOD, CAT, HO-1, NOS, COX-2, GSH), which also limit the lipid peroxidation of low-density lipoprotein (LDL) cholesterol, a hallmark of atherosclerosis. Lowered inflammation and oxidative stress and an improved lipid profile were also demonstrated in healthy subjects as well as in metabolic syndrome patients after hydroxytyrosol (HT) supplementation. These results might open a new therapeutic scenario through personalized supplementation of HT in CVDs. This review is the first attempt to collect together scientific literature on HT in both in vitro and in vivo models, as well as in human clinical studies, describing its potential biological effects for cardiovascular health.

## 1. Introduction

According to the World Health Organization (WHO), cardiovascular diseases (CVDs) are the first cause of death globally and have also led to an elevated economic impact. CVDs comprise several disorders, such as heart failure, hypertension, coronary artery disease, peripheral vascular disease, congenital heart disease, and stroke [1,2]. Lack of exercise, overweight and obesity, diabetes, stress, alcohol consumption, aging, smoking habits, and unhealthy dietary patterns are all responsible for increased CVD risk [3,4,5]. A large number of studies have clearly established that the Mediterranean (Med) diet is the cornerstone of CVD prevention. In Med diet, virgin olive oil (VOO) and extra-virgin olive oil (EVOO) are the major sources of fat and active phenolic compounds, responsible for the minor mortality and morbidity noted in people on such a diet [1,6,7]. Besides high levels of monounsaturated fatty acids (MUFA), VOO and mostly EVOO contain several minor phenolic components with biological properties, such as the ability of modulating biomarkers involved in pathways implicated in the development of atherosclerosis. Atherosclerosis may be considered an inflammatory disease, characterized by the accumulation of macrophage-derived foam cells in the vessel wall with the production of cytokines, chemokines, and growth factors, together with oxidative stress. In this scenario, blood vessels are damaged, and their remodeling leads to blood flow restriction, responsible for heart and nervous system injury [8]. Among the phenolic compounds present in EVOO, hydroxytyrosol (HT) was reported to counteract CVD, owing to a wide range of biological activities, such as prevention of endothelial dysfunction and macrophages activation, in turn limiting low-density lipoprotein (LDL) cholesterol oxidation, modulation of the blood lipid profile, reduction in platelet aggregation and reduction in chronic inflammation. [9,10,11]. This review describes the vast range of known biological information about HT, focusing on its involvement in biological mechanisms of CVD. It is the first attempt to collect the scientific literature on the effects of HT in both in vitro and in vivo models and also in human clinical trials.

## 2. Methodology

A systematic literature search using PubMed and Web of Science databases was carried out to summarize the biological activities of HT in cardiovascular diseases. The original articles were individuated, employing the keywords: “hydroxytyrosol” AND “oxidative stress” OR “anti-inflammatory” OR “bioavailability” OR “metabolism” OR “cardiovascular disease” OR “in vitro study” OR “in vivo study”, including the period 2000–2020. This literature was beyond manual choice in agreement to the importance to the focus. Literature from before the year 2000 was faithfully selected and inserted when indispensable considering its importance to the reviewed topic. Studies on crude olive extract or a mixture of olive polyphenols were excluded from this review.

## 3. Biochemical Properties of Hydroxytyrosol

**Figure 1:**
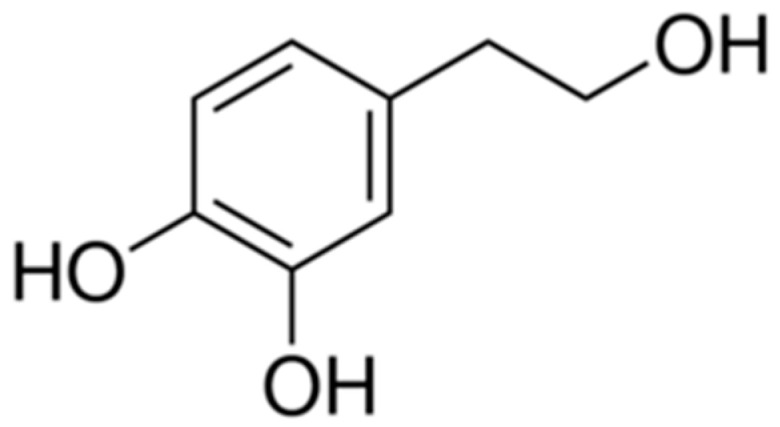
Chemical Structure of hydroxytyrosol (HT).

HT is an amphipathic phenol with a phenyl-ethyl-alcohol structure (Figure 1) and is part of the soluble fraction of extra virgin olive oil (EVOO).

Due to the amphipathic character of HT, it can be found in olive mill wastewater, pomace, and olive oil, on a free form, as acetate form or as a component of other compounds like oleacein, verbascoside, tyrosol, and oleuropein [12,13]. HT is obtained from the hydrolysis of oleuropein which happens naturally during the ripening of the olives, and with the over-time oil storage [14]. The content of HT in olive oil is determined by the kind of olive tree, the location of the plantation, the oil quality, and the olive oil elaboration process [12].

HT effectiveness in vivo should be carefully evaluated considering the kinetics of absorption and metabolism of this compound after ingestion. Many studies have been performed in animals, and fewer in humans, in order to determine how adsorption, distribution, metabolism, and excretion of HT occurs. Similar to other phenolic compounds, HT is quickly absorbed in the small intestine and colon, mainly by passive transport, in a dose-dependent manner, with efficiency ranging from 75% up to 100% [15,16,17]. HT and all polyphenols are exposed to an extensive intestinal/hepatic metabolism in the human body, and their bioavailability is poor in plasma compared to their metabolites [17]. After ingestion, HT undergoes phase I of metabolism, being firstly hydrolyzed in enterocytes and subsequently, through phase II of metabolism, it is metabolized into glucuronide and methylated and sulphate bio-products [18,19]. After these processes, it is possible to detect 98% of HT in glucuronide form, in plasma and urine, and only 2% as its free form, when it is administered in olive oil [20].

HT has a fast metabolism and its estimated plasma half-life in rats has been reported to be about 2 min [21,22] and 8 min in human healthy volunteers [23]. A while ago, the maximum plasma concentration was reported to be around 7 min after intake in rats [24]. In spite of this, other authors have reported that the highest plasma concentrations of HT are reached between 30 min and 2 h after its oral administration, being practically undetectable after 4 h in rats [25]. In humans, the maximum concentration is detected after 13 min, decreasing till undetectable levels 1 h after administration [23]. In fact, once absorbed, HT quickly becomes part of plasmatic lipoproteins, acting as an antioxidant and as a cardiovascular protector [26]. González-Santiago and coworkers, demonstrated in a group of healthy volunteers, that about 70% of the total HT orally supplemented in aqueous solution, was detected in plasma purified LDL, the concentration was higher after 10 min from the HT intake and lower after 20 min, due to the fast HT elimination from the plasma [23].

In spite of the short plasma half-life, using Wistar rats as an in vivo model, HT and its metabolites demonstrated the ability to good distribute in skeletal muscles, testis, heart, liver and also in the brain, considering that HT is able to cross the blood–brain barrier, besides its metabolism ends in the liver and in kidneys [15,21,27]. Thanks to this widespread distribution, HT exerts its health-beneficial properties in all body tissues. In the kidney, HT is accumulated until its main excretion as conjugated catabolites, still performing there a nephroprotective role due to its antioxidant properties [21,27,28]. The time required for the complete elimination from the body, both for HT and its metabolites, is approximately 6 h in humans [18,29] and around 4 h in rats [25]. Adsorption and excretion processes for HT depend not only on the in vivo model used but can also be influenced by the vehicle employed for the administration [15,30].

## 4. Biological Effects of Hydroxytyrosol in Cellular In Vitro Models

Many studies have been carried out with different cellular models in order to observe the beneficial effects of HT and to try to understand the underlying molecular antioxidant and anti-inflammatory mechanisms and the anti-atherogenic action of the compound. All the biological activities of HT in cellular in vitro models, directly related to atherosclerosis and CVD risk, are summarized in Table 1.

### 4.1. HT as Radical Species Scavenger: Protective Role against Oxidative Stress Contributing to Atherosclerosis

The induction and progression of CVD have been related to the disturbance of cellular redox balance, characterized by increased levels of reactive oxygen species (ROS) and reactive nitrogen species (RNS), such as superoxide anion (O_2_^−^), hydrogen peroxide (H_2_O_2_), hydroxyl radical (OH^·^) and peroxynitrite (ONOO^−^) [31].

ROS are products of normal cell activity and participate in cellular signaling. However, high levels of ROS have dangerous effects on cellular homeostasis and functions, resulting in oxidative stress and endothelial dysfunction in vessel walls [32]. Therefore, lowering ROS production and restoring cellular antioxidant defenses could prevent atherosclerosis risk. The superoxide dismutase 1 (SOD1) enzyme catalyzes the transformation of two superoxide anions in a molecule of H_2_O_2_ and oxygen (O_2_). After that, the detoxification initiated by SOD is completed by the catalase (CAT) enzyme, which converts H_2_O_2_ to water and oxygen [31]. Glutathione peroxidase (GPx) is a selenium-containing enzyme, which also catalyzes the degradation of H_2_O_2_, as well as organic peroxides to alcohol.

RNS are a family of nitrogen moieties associated with oxygen. They are generated when nitric oxide (NO) interacts with reactive oxygen species, such as O_2_^−^ and H_2_O_2_. Enzymatic NO formation is catalyzed by NO synthase (NOS) enzymes (eNOS—endothelial, nNOS—neuronal, and iNOS—inducible) through a series of redox reactions. The cardiovascular roles of NOSs have been widely studied, and the findings supply significant insights into the importance of NOSs in CVD. Overall, eNOS and nNOS exert protective effects, while iNOS has dual roles in the cardiovascular system. NO is a key molecule involved in a variety of biological functions throughout the whole body [33]. In the vasculature, NO (major part from eNOS, but nNOS is present around arterioles) plays an important role in the protection against the onset and progression of CVD [34]. In the presence of oxidative stress, NO reacts quickly with O_2_^−^ producing the highly reactive intermediate OONO^−^, which causes severe damage in the endothelial tissue due to the uncoupling of the eNOS enzyme and reduction in NO bioavailability [35], a hallmark of CVD.

**Figure 2:**
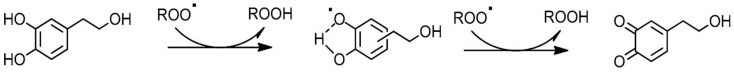
Mechanism of free radical scavenging exerted by hydroxytyrosol (HT).

HT has a strong antioxidant activity, due to its high capacity to eradicate both the intracellular and extracellular production of ROS, being mainly effective with free radical molecules, such as O_2_^−^ and H_2_O_2_, acting as a metal chelator as well [36]. These properties are due to both the presence of hydroxyl (OH) groups in ortho position, who have electron donating capacity, and to the HT ability to bind phenoxyl radicals, forming stable hydrogen bonds (Figure 2) [37,38].

The antioxidant effect of HT does not depend only on the capacity of scavenging oxidant chemical species, but also on the ability to stimulate the synthesis and activity of antioxidant enzymes SOD, CAT, NOS, GPx and glutathione reductase (GR), also preserving the cellular high levels of reduced glutathione (GSH) [39,40].

Diminished NO availability has been related to the development of atherosclerosis, and the upregulation of eNOS activity could represent a strategy for the prevention of vascular complications. In a study performed on human endothelial cells (EA.hy926) without inflammatory conditions, no evidence was obtained after HT treatment (0.1–100 µM) on eNOS gene promoter transactivation, eNOS enzyme activity and NO availability [41]. Nevertheless, in a recent paper using hydroxytyrosol-nitric oxide (HT-NO), an experimental drug, in which HT was combined with NO, demonstrated antioxidant and NO-releasing activities. HT-NO increased NO levels and decreased oxidative stress activating deacetylase Sirtuin 1 (SIRT1) expression, in high glucose-stimulated human umbilical vein endothelial cells (HUVECs). The ROS scavenger HT enhanced the effect of HT-NO on eNOS phosphorylation [42]. HT was also investigated in ECV304 cells, a cellular model of endothelial dysfunction in the type 2 diabetes condition. In this study, HT treatment (10 μM for 48 h) induced a significant up-regulation of endothelin-1 (ET-1) and eNOS phosphorylation, as well as NO production, being effective in the protection against endothelial dysfunction, induced by high glucose and free fatty acids [43].

During oxidative stress, polyunsaturated fatty acids, present in the membranes of endothelial and smooth muscle cells in blood vessels, are oxidatively damaged. Lipid peroxidation, especially referring to LDL, has been reported to have an important role in the progression of atherosclerosis. Oxidized LDL (oxLDL) is cytotoxic and chemotactic, and monocyte macrophages avidly remove oxLDL from the interstitium, generating macrophage foam cells, the major cell type present within fatty streaks and fibrous plaque [44].

The antioxidant effect of HT in counteracting LDL oxidation induced by copper sulphate is not recent in the scientific literature, demonstrating the reduction in lipid peroxidation markers of F2-isoprostanes, as well as a reduced decline in vitamin E throughout LDL oxidation in vitro [45,46].

Enriching LDL and HDL with tyrosol and HT (up to 25 μM) led to a major resistance to lipid peroxidation, also favoring the reverse cholesterol transport. Cholesterol efflux from THP-1 cells-derived macrophages was intensified, probably repressing the effect of Fe/Asc on the cell surface receptors implicated in this process. Tyrosol and HT stimulated ATP binding cassette subfamily A member 1 (ABCA1) protein expression in J774 macrophages, promoting the efflux of phospholipids and cholesterol efflux to lipid-poor apoA-1, through a system involving the direct binding of apo-AI to the ABCA1 transporter [47]. Another study by Atzeri et al. in 2016 showed the antioxidant role of HT in neutralizing the pro-oxidant effect obtained treating Caco-2 human enterocyte-like cells with oxidized cholesterol. Caco-2 cells, exposed to HT (5–25 μM), had significantly blocked malondialdehyde (MDA) increase, inhibited ROS production, and induced glutathione peroxidase activity, whilst maintaining GSH levels and cell viability [48]. These results may be due to the antioxidant effect of the phenolic compound HT, which may scavenge ROS and therefore prevent lipoprotein oxidation.

In vitro studies carried out on human neutrophils evidenced that the biophenol HT (up to 50 μM) was able to remove N-formyl-methionyl-leucylphenylalanine (fMLP), phorbol myristate acetate (PMA), and opsonized zymosan-induced injury, which is mediated by H_2_O_2_ in oxidative conditions [49].

Imbalance in mitochondrial functions, with production of higher levels of mitochondrial ROS (mtROS), may also contribute to endothelial dysfunction and CVD. The endothelial protective effect of HT occurs, also reducing mitochondrial superoxide production by improving mitochondrial function and biogenesis. The HT pretreatment of PMA-activated endothelial cells reduced O_2_^−^ production and membrane lipid peroxidation as well as reduced mitochondrial membrane depolarization, preventing mtROS overproduction. Moreover, HT increased mtDNA content and the master regulator of biogenesis the Peroxisome proliferator-activated receptor gamma coactivator-1α (PGC-1α), the transcription factor Nuclear respiratory factor-1 (NRF-1), and Mitochondrial transcription factor A (TFAM) gene expression, resulting in an improved mitochondrial functionality [50].

In H9c2 cardiomyocytes with increased activity of xanthine/xanthine oxidase (X/XO), which represent a risk factor for heart disease, HT treatment (0.1 and 10 μg/mL, for 24 h) induced a reduction in intracellular ROS level and a modulation of stress-sensitive pathways across the upregulation of defensive proteins, p44/42-MAPK and Hsp27 via c-Jun [51]. In porcine pulmonary artery endothelial cells treated with H_2_O_2_, HT (10, 30 and 50 μM) prevented the intracellular overproduction of ROS and induced AMP-activated protein kinase (AMPK) as well as forkhead transcription factor 3a (FOXO3a), triggering the upregulation of the antioxidant enzyme CAT [52].

The protective effects of HT have also been reported on human erythrocytes, in which the HT treatment reduced ROS production and increased GSH level, preventing haemolysis and morphological alterations, induced by exposure to HgCl_2_ [53]. Another study highlighted that HT pre-treatment (10, 25 and 50 μM) reduced erythrocyte phosphatidylserine (PS) exposure at the cell surface, and re-estabilished ATP and GSH amount, indicating that HT could also take part in modulating the programmed death in non-nucleated cells [54].

The HT antioxidant beneficial effects could also depend on the activation of signaling pathways involved in the recognition of the free radicals’ presence. In fact, in human retinal pigment epithelial cells, from the ARPE-19 cell line, 100 μM HT was demonstrated to modulate the expression of several genes encoding for antioxidant response elements (ARE), such as DNA-repair proteins or phase II detoxifying enzymes, inducing the nuclear factor-E2-related factor-2 (Nrf2) and JNK-p62/SQSTM1 pathway [55]. The HT induced synthesis and translocation of Nrf2, and the promotion of phase II detoxifying enzymes, such as heme oxygenase 1 (HO-1), was also demonstrated by Zrelli et al. in 2011 and 2015, with particular interest in the atheroprotective effects of HT (50 μM) [56]. Zrelli and coworkers showed the HT-mediated upregulation of HO-1 activity and increased cell proliferation and wound closure on an in vitro model of a wound using cultured vascular endothelial cells (VECs). HT was also effective in protecting VECs from oxidant-induced injury, stimulating the Nrf2 via PI3K/Akt and ERK1/2 pathways [56,57].

### 4.2. Anti-Inflammatory Role of HT

Inflammation and the activity of the immune system are classically considered beneficial and protective. In fact, inflammatory mediators, such as cytokines, chemokines, RNS, and ROS, released directly by the immune system cells or by activated endothelial cells, can attract monocytes, T- and B-lymphocytes, to the injured site, with the purpose of clearing infection and removing debris while initiating tissue repair mechanisms [58,59]. The inflammatory response, generally quick and self-limiting, is safe for the host, but may also have a detrimental effect if becoming a chronic low-grade inflammation, as inevitably associated with older age, obesity and visceral adiposity, triggering the prodromal stage of CVD, dementia, autoimmune diseases, and many more. In this condition endothelial, smooth muscle, and peripheral blood cells are chronically exposed to high levels of potentially toxic molecules, proinflammatory cytokines, chemokines, eicosanoids (prostaglandins and leukotrienes) and autoantibodies [60,61].

HT, tested in a concentration range from 12.5 to 50 μM, greatly reduced the expression of iNOS, Cyclooxygenase-2 (COX-2), prostaglandin-endoperoxide synthase 2 (PTGS2), chemokines (CCL5/RANTES, CXCL10/IP10, and CCL4/MIP1β), Interleukin-1α (IL-1α), and Matrix metallopeptidase-9 (MMP-9) genes in the RAW264.7 macrophage cell line, previously stimulated with the proinflammatory molecule LPS (lipopolysaccharides). The higher concentration of HT 50 μM was more effective as an anti-inflammatory compound acting through Nuclear Factor-κB (NF-κB) inhibition [62]. In human monocytic THP-1 cell lines, the HT molecular mechanism of action appeared to be related to a crucial decrease in protein kinase C (PKC)-β1 and PKCα membrane translocation, inhibiting proinflammatory cytokine production and the activation of iNOS [40]. Additionally, in PMA-activated U937 human monocytes, 1–10 μmol/L of HT was able to reduce MMP-9 and COX-2 expression and activity through the inhibition of the nuclear translocation of NF-κβ and of PKCα and PKCβ1 activation [63]. In a study from Takeda et al. (2014), it was shown that HT may also repress NO production by a mechanism independent of the NF-κβ pathway [64]. Rosignoli et al. performed an in vitro experiment on isolated human monocytes. Their data demonstrated that HT (100 μM) significantly inhibited the production of superoxide anions (O_2_^−^), and reduced COX-2 expression and PGE2 release [65]. Moreover, in J774 murine macrophages, HT also down-regulated proinflammatory molecules, iNOS and COX-2, counteracting NF-κB, STAT-1α and IRF-1 activation, mediated through LPS-induced ROS generation [39].

Data from these in vitro studies offer a molecular basis for clarifying the beneficial preventive effects of HT on inflammatory signaling involved in atherosclerotic processes.

### 4.3. Modulation of Endothelial and Macrophage Activation in Protection from Atherosclerosis

Endothelial dysfunction is the prodromal stage of atherosclerosis, which exposes the individual to an increased risk of CVD onset. E-selectin, P-selectin, Vascular Cell Adhesion Molecule-1 (VCAM-1), Intercellular Adhesion Molecule-1 (ICAM-1), and the Monocyte Chemotactic Protein-1 (MCP-1) molecules, can be considered as biomarkers of endothelial and immune cells dysfunction. In fact, the leukocytes rolling on the vessels’ endothelial wall is mediated by E and P-selectin expression by the activated endothelial monolayer that, subsequently expressing VCAM-1and ICAM-1, favors the leukocyte adhesion. Leukocyte migration into the intima is then mediated by MCP-1 release.

The inhibition of endothelial activation by free HT was described in the HUVEC cell line, observing that HT was able to reduce the LPS-stimulated expression of VCAM-1 at low micromolar concentrations. The reduction in E-selectin, VCAM-1, and ICAM-1 expression was observed in HUVEC after HT exposure at doses between 5 and 25 μM [66]. In a study performed on human endothelial cell line EA.hy 926, HT treatment (0.5–2.5 μM) significantly reduced monocyte adhesion and ICAM-1 expression, induced by homocysteine [67]. In murine RAW264.7 macrophages cultured with 25 μM HT, a significant reduction in MCP-1 was observed [62,68]

The reduction in MCP-1 and the increase in SOD1 antioxidant enzyme, were achieved in an interesting paper using HUVEC treated with human serum, collected from recruited subjects, after their intake of VOO with the high content of phenolic compounds. Carluccio et al. demonstrated that the HT ability in counteracting endothelial inflammation and activation is mediated by the inhibition of intracellular ROS and the NF-κB pathway, in the inflamed endothelium [69]. The treatment of cultured HUVEC with serum obtained after the intake of the high-phenol VOO-based breakfast in human healthy volunteers, significantly decreased p65, MCP-1, and CAT gene expression and increased MT-CYB, SDHA and SOD1 gene expression levels [70]. Thus, decreasing inflammation and improving the antioxidant profile in the vascular endothelium, olive oil phenolic compounds could reduce atherosclerosis risk. Moreover, considering that, after the consumption of normal doses of VOO (less than 50 g/day), the native form of HT is not detected in plasma, the potential health benefits of VOO phenolic compounds could be either attributed to the biological activity of HT metabolites [71]. Interestingly, cultured Human Aortic Endothelial Cells (HAEC) in the presence of Tumor Necrosis Factor-α (TNFα) stimulation and synthetized HT metabolites, obtained in culture by intestinal Caco-2 cell line, resulted in the effective reduction in E-selectin, P-selectin, VCAM-1, and ICAM-1 [71].

### 4.4. Antithrombotic Effect

Among the processes involved in CVD and atherosclerosis, platelet aggregation must be considered. HT showed anti-thrombotic activities, as it considerably diminished platelet aggregation both in vitro and ex vivo, reducing thromboxane B2 levels, a chemically stable and inactive form of thromboxane A2, NO and leukocyte inflammatory mediators [72,73]. In a study from Dell’Angi et al., human platelets collected from healthy donors and stimulated with thrombin were used for an aggregation assay ex vivo. Different olive oil polyphenols, including HT, showed the inhibition of platelet aggregation through cAMP-phosphodiesterases inhibition as a mechanism of action [72].

Reyes and coworkers, in order to increase the lipophilicity of HT, synthetized HT alkyl ether derivatives and tested the in vitro antiplatelet aggregation effect of the compound, compared to HT tested in human whole blood. HT alkyl ether derivatives exerted greater antiplatelet aggregation and anti-inflammatory effects than HT. This effect was explicated through a slight reduction in platelet thromboxane synthesis, an enhancement in constitutive NO production and inhibition of iNOS as well as COX-2 enzymes [74].

### 4.5. Antiadipogenic Role

Local and systemic chronic low-grade inflammation with increased oxidative stress, are typically present in obesity and play a key role in both CVD and type 2 diabetes mellitus. The major amount of adipose tissue in obesity is characterized by an aberrant production of pro-inflammatory cytokines, among which TNFα, derived from both adipocytes and infiltrating macrophages, and adipose tissue specific adipokines [8,75]. Scoditti et al. demonstrated the stimulatory effects of HT on adiponectin, after the TNFα-induced downregulation of this adipokine expression, in cultured adipocytes. Indeed, adiponectin, is an adipocyte-derived insulin-sensitizing and anti-inflammatory hormone, that is reduced in obesity through mechanisms involving chronic inflammation and oxidative stress, as well as with anti-diabetic, anti-inflammatory, and anti-atherogenic properties [75,76].

Molecular studies have also shown that HT might control the expression of adipogenesis-associated genes. HT-treated primary human omental pre-adipocytes significantly increased the expression of GATA2, GATA3, WNT3A, SFRP5, HES1, and SIRT1 genes involved in adipogenesis suppression. Genes involved in promoting adipogenesis, such as LEP, FGF1, CCND1, and SREBF1, a transcription factor necessary for lipogenesis, were significantly down-regulated. Moreover, HT treatment was able to reduce triglyceride accumulation in adipocytes, suggesting a lipolytic and apoptotic activity in primary human visceral pre-adipocytes during differentiation, regulating the expression of genes involved in adipogenesis and fat storage pathways [77]. These achievements demonstrate that HT can have a protective role against fat accumulation and obesity and, therefore, can be useful in preventing diseases caused by these factors.

## 5. Health Beneficial Effects of Hydroxytyrosol Demonstrated in Animal In Vivo Models

To better elucidate the effects and the molecular mechanisms of the HT protection against LDL oxidation, inflammatory response and macrophages and endothelial activation, as well as obesity, hyperlipidemia and cancer, in vivo animal models were largely used. The HT administration to in vivo animal models allowed the investigation of the potential healing role of the compound in a more complex picture, mimicking the pathological condition. A summary of relevant studies on HT supplementation in animal models is reported in Table 2.

### 5.1. Protection of Low-Density Lipoprotein (LDL) from Oxidation

Lipid peroxidation, especially referred to oxidative modified LDL, has been reported to have a key role in atherosclerotic plaque formation. A minor susceptibility to lipid peroxidation and particularly to LDL oxidation was found significant in a rabbit model fed with an olive oil rich diet [78]. Using Wistar rats fed a cholesterol-rich diet for 16 weeks demonstrated the significant reduction in lipid peroxidation in liver, heart, kidney, and aorta, specifically due to HT orally administered to rats, compared with those animals fed only the cholesterol-rich diet. This antioxidant activity could be explained with increased CAT and SOD enzymatic activities observed in the liver of this hypercholesterolemic rat model [79,80]. HT supplementation was also effective in decreasing the oxidation levels of lipids and proteins in both liver and muscle tissue. This effect was obtained decreasing muscle mitochondrial carbonyl protein levels and thus improving mitochondrial complex activities in C57BL/6J mice model, in which obesity and oxidative stress was induced through high-fat-diet [81]. HT also improved the cardiac disturbances enhanced by doxorubicin in rats, by significantly reducing the percentage of altered mitochondria and oxidative damage and improving the mitochondrial electron transport chain [22]. The antioxidant role of HT was also supported by its protective effect against the NO-mediated vasorelaxation after the induction of oxidative stress in rat aorta [82]. Excised rat aorta pre-incubation with HT, before treatment with cumene hydroperoxide, an oxidative injury inducer through OH^·^ generation, preserved the vasodilation mediated by NO, recovering the aortic vascular tone ex vivo. This effect was attributed to high scavenging activity of HT due to the presence of a catechol moiety in their structure [82]. These results suggested that HT could be a natural antioxidant with a possible role in the prevention of atherosclerotic lesions and cardiovascular events.

### 5.2. Improvement of Blood Lipid Profile

Plasmatic hyperlipidemia is the result of changes in lipid metabolism in the body and is a major cause of atherosclerosis. Hypercholesterolemia, or more specifically increased plasma levels of LDL cholesterol, is an important risk factor for the development and progression of atherosclerosis. The administration of HT (3 mg/kg of body weight) decreased significantly the serum levels of total cholesterol (TC), triglycerides (TG), and LDL and increased the serum level of high-density lipoprotein (HDL) in a diet-induced hypercholesterolemic rat model [79]. These results are in accordance with a previous study, in which lower dose of HT (2.5 mg/kg of body weight) significantly reduced the serum levels of TC and LDL, while increasing HDL, in the same rat model [80]. Recently, the comparative evaluation between HT, HT-acetate, and HT-ether supplementation in diet-induced hypercholesterolemic Wistar rats, showed improved cholesterol, glucose, insulin, and leptin levels together with an increased antioxidant capacity status, with HT-acetate being the most effective compound [83].

In a rabbit model of atherosclerosis, induced with 1-month of a high-fat diet containing 2.5 times more saturated fat and 1.3% more cholesterol than a standard diet, supplementation with 4 mg/kg of purified HT improved the blood lipid profile, the antioxidant status also reduced the size of the atherosclerotic lesions histologically evaluated [84]. Within liver and skeletal muscle tissues, HT effectiveness was demonstrated to reduce lipid deposits due to a high-fat-diet through the inhibition of the SREBP-1c/FAS pathway. These achievements were obtained after 17 weeks of HT supplementation in C57BL/6J mice, in which obesity and hyperlipidemia was induced through a high-fat-diet [81]. These results suggested that HT administration might improve the blood lipid profile and obesity, reducing the risk of atherosclerotic lesions.

### 5.3. Hypoglycemic Ability

Cao and coworkers also potentially linked the olive oil component HT to diabetes and metabolic disease treatment. They demonstrated that HT could prevent obesity, hyperglycemia, and insulin resistance, after 17 weeks of supplementation in db/db mice, a model of metabolic syndrome (MetS) with obesity and type 2 diabetes induced by the nonfunctional leptin pathway [81]. In this model, 10 mg/kg/day of HT significantly decreased fasting glucose, similar to 225 mg/kg/day metformin. Moreover, HT was also able to decrease fasting serum levels of TC, HDL, and LDL, at which metformin failed [81]. In streptozotocin-induced diabetic mice, 77 mg/Kg of HT significantly lowered blood glucose, at 4 weeks [42]. HT health benefits could not be limited to decreases in oxidative stress, suggesting a potential pharmaceutical or clinical use of HT in MetS treatment.

### 5.4. Anti-Inflammatory Activity

The excessive deposition of adipose tissue with adipocytes dysregulated the production of pro-inflammatory cytokines, such as TNFα, IL-1β, IL-6, leptin, and adiponectin, which might participate in the development of insulin resistance, endothelial dysfunction and atherosclerosis in obese patients and in the associated MetS [8]. Serum IL-6 and C-reactive protein (CRP) levels were significantly increased in high fat diet-induced obese mice, demonstrating an increased inflammation in obesity condition. Both low and high doses of HT (10 mg/kg/day and 50 mg/kg/day, respectively) efficiently decreased the elevated IL-6 level, and high-dose HT treatment diminished the CRP level by 22.4% [81]. Diet supplementation with HT, HT-acetate, and HT-ether in Wistar rats fed with cholesterol-rich diets, showed anti-inflammatory effects, decreasing the plasma level of TNFα and IL-1β pro-inflammatory cytokines. Moreover a significant decreased in MCP-1 and IL-1β below the control levels (group of rats fed standard rodent diet without HT supplementation) was detected in the visceral adipose tissue from the retroperitoneal area of this rat model, supplemented with HT [83].

### 5.5. Antithrombotic Effect

Platelet aggregation is one of the main events occurring in arterial and venous thrombosis and HT inhibitor effect on platelets aggregation could be potentially useful in new therapeutical approaches to CVD. González-Correa et al. compared the effect of orally administered HT and its main metabolite HT-acetate, with that of acetylsalicylic acid, chosen because of its wide use in ischemic CVD prevention. All three compounds studied (48.25 mg/kg per day for HT, 16.05 mg/kg per day for HT-acetate, compared to 2.42 mg/kg per day of acetylsalicylic acid) inhibited collagen-induced platelet aggregation in whole blood and in a dose-dependent way, in Wistar rats [85]. Moreover, the platelet synthesis of thromboxane B2 was inhibited by up to 30% by HT and 37% by HT-acetate.

## 6. Health Beneficial Effects Demonstrated Through Clinical Trials in Humans

The availability of clinical studies in humans, considering the effects of VOO and EVOO is wide and the PREDIMED study (Prevención con Dieta Mediterránea), conducted in Spain from 2003 to 2011, made a big effort to assess the effects of the Med diet on the prevention of atherosclerosis and CVD [86,87,88], and MetS [89,90]. Although the incidence of cardiovascular events was lower among patients who followed a Med diet regimen integrated with EVOO, no further investigations were conducted in the PREDIMED study and in its subsequent sub-studies, on the specific effect of the olive oil polyphenols, on CVD risk. The NUTRAOLEUM study evaluated the effect of VOO enriched with phenolic compounds and triterpenes, on MetS and on biomarkers of endothelial function in healthy adults. The daily intake of VOO high in phenolic compounds ameliorated plasma HDL cholesterol levels, one of the features of MetS. In addition, VOO with phenolic compounds improved the systemic endothelin-1 levels in vivo and ex vivo [91,92,93]. Studies about purified polyphenols and HT supplementation in humans are still limited. However, in 2011, the European Food Safety Authority (EFSA) endorsed the health claim on HT consumption for the protection of blood lipids from the oxidative damage [94].

Table 3 elaborates on the available scientific literature about HT supplementation in human clinical trials.

### 6.1. Protection of Low-Density Lipoprotein (LDL) from Oxidation

Well-designed studies contributed to the claim of HT as antioxidant compound for circulating LDL by EFSA; among these, we reported on the multicenter scientific work of Covas et al. in 2006 [95]. In the study, 200 healthy male volunteers were randomly assigned to three sequences of daily administration of 25 mL of three different olive oils containing 2.7 mg, 164 mg, or 366 mg per kg of oil of phenolic compounds. The intervention periods was 3 weeks. A linear decrease in oxLDL for low-, medium-, and high-polyphenol olive oil with more than a 3-fold decrease for higher concentrations of polyphenols, was observed [95]. Considering the same quantity of oral intake of phenolic compounds in olive oil (366 mg/kg, 164 mg/kg, and 2.7 mg/kg), Covas and coworkers also observed a modulation in the LDL phenolic content and a reduced postprandial oxidative stress, in 12 healthy male volunteers. The higher the phenolic content of the olive oil administered, the lower the resulting LDL oxidation level [96].

Interesting results were also obtained in 40 male subjects with stable coronary heart disease, in a placebo controlled, crossover, randomized trial [37]. Using EVOO and refined olive oil with polyphenol contents 161 mg and 14.7 mg per kg of oil, respectively, significant lower plasma levels of oxLDL, together with higher activities of glutathione peroxidase, were observed after EVOO intervention. Additionally, the systolic blood pressure significantly decreased after the intake of EVOO, in these hypertensive patients [37].

A more recent randomized, double-blinded, placebo-controlled, crossover trial was performed for 20 weeks by Quirós-Fernández et al. in 2019, demonstrating the effectiveness of 9.9 mg HT daily supplementation, together with 195 mg of punicalagin (from pomegranates), on early atherosclerosis markers. In 84 recruited subjects, aged between 45 and 65 years, the oral supplementation of compounds increased endothelial function in flow-mediated dilatation, reduced oxLDL, and also decreased the systolic and diastolic blood pressure, compared to placebo [97]. The modulation of these early atherosclerosis markers was especially effective in individuals in whom these parameters were altered.

A significant decrease in nitrite, nitrate, and MDA, as well as an increase in thiol groups, used as oxidative stress biomarkers, were obtained after 15 mg/day HT supplementation, in healthy volunteers, recruited in a clinical trial from Colica and coworkers in 2017 [98]. A significant upregulation of SOD1 gene expression was also detected, without, however, observing a reduction in oxLDL levels. These obtained results were positively correlated with the significant increase in HT bioavailability [98].

### 6.2. Modulation of Blood Lipid Profile

The multicenter scientific work of Covas et al. in 2006 also demonstrated a linear increase in HDL cholesterol levels for low-, medium-, and high-polyphenol olive oil, after 3 weeks administration in 200 healthy males [95]. TC–HDL ratio decreased linearly with the phenolic content of the olive oil. Triglyceride levels decreased by an average of 0.05 mmol/L for all types of administered olive oils [95]. No significant changes were observed in healthy volunteers, after HT supplementation in oral capsules (15 mg/day) for a 3 week period, observing peripheral triglycerides, TC, and HDL levels. However, a significant reduction in body weight and suprailiac skinfold were both reported after HT supplementation with respect to baseline [98].

### 6.3. Increased Insulin Sensitivity

Very limited data are available on the possible effects of HT and olive oil polyphenols on glucose homeostasis in humans. A randomized, double-blinded, placebo-controlled, crossover trial was well designed in New Zealand, supplying 46 middle-aged overweight men, with capsules containing 51.1 mg oleuropein and 9.7 mg HT, or placebo, per day for 12 weeks [99]. The primary outcome was insulin sensitivity, which had improved by 15% together with the increased pancreatic β-cell secretory capacity, in recruited overweight middle-aged men, possibly reducing the risk of developing the MetS [99].

### 6.4. Anti-Inflammatory Activity

Recently, a sub study of 1139 CVD high-risk participants was carried out within the PREDIMED (Prevención con Dieta Mediterránea) trial, demonstrating a significant inverse correlation between total urinary polyphenol excretion (TPE) and plasma concentration of VCAM-1, ICAM-1 and inflammatory biomarkers IL-1β, TNFα, and MCP-1, suggesting a dose-dependent anti-inflammatory effect of polyphenols. Instead, a positive correlation was found between plasma HDL cholesterol and urinary TPE levels [88]. However, in the scientific study, specific considerations about the HT compound were not found. Camargo et al. observed that VOO in vivo (HT content 0.2 μmol g^−1^ and 45.4 μmol g^−1^, respectively in low-phenol and high-phenol olive oil) was able to downregulate the expression of several genes related to inflammation pathways in patients with MetS, during the postprandial period. A decreased expression of IL-6, IL-1β, PTGS2, and diverse chemokines, as compared to low-phenol olive oil intake, was detected. Proinflammatory stimuli, such as IL-6, IL-1β and TNFα cytokines, up-regulate PTGS2 expression and thus COX-2 and prostaglandin synthesis, indeed chronically influencing the inflammatory condition [100]. The authors described these anti-inflammatory effects as a consequence of phenol interaction with the NF-κB/MAPK/AP-1 signaling pathways. Thus, the anti-inflammatory effect of HT contributes to the atheroprotective properties of olive oil, suppressing the iNOS/NO and the COX/PGE2 pathways, leading to a minor atherosclerotic plaque instability and risk reduction in acute cardiovascular events [101].

### 6.5. Antithrombotic Effect

Ruano and coworkers evaluated the effects of olive oil phenols on endothelial reactivity, also considering that endothelial-dependent vasodilatation and oxidative stress are impaired during the postprandial phase, favoring a thrombogenic state. In 21 hypercholesterolemic middle-aged volunteers, the same olive oil was administered during breakfast meals, but with a different content of its natural phenolic compounds, 400 ppm or 80 ppm. Polyphenol-rich oil was associated with a significant increase in NO levels and improving endothelium functions and microvascular vasodilatation 2 h after the breakfast intake [102]. Moreover, 2 h after the high-phenol meal, concentrations of activated factor VII (FVIIa) increased less, and plasmatic plasminogen activator inhibitor-1 (PAI-1) activity decreased more than after the low-phenol meal. The VOO with the higher content of phenolic compounds modulated the postprandial hemostatic profile, making it less thrombogenic. However, in both studies, the phenolic composition of the olive oils used was not properly characterized [103].

## 7. Conclusions

In this review, we have reported on the pleiotropic effects of HT in CVD prevention, and we have strengthened the relationship between inflammation, obesity, oxidative stress, and diet, providing evidence of healthy effects derived from VOO consumption and HT. The scientific data available to date are encouraging, and the use of HT also in the form of a dietary supplement seems promising as an adjuvant of drug therapy. However, it would be necessary to evaluate how longer HT beneficial effects are maintained after feeding and to better characterize the effects carried out by the several olive oil phenolic compounds, in order to understand if they act synergistically. Moreover, even if 5 mg/die is the minimum HT dose recommended by EFSA, the HT doses used in the wider literature here reported would suggest the need for personalized HT supplementation, in order to exert its health benefits in CVD prevention. Thus, further human clinical trials are needed, with a larger population over a longer period, in order to increase knowledge about therapeutic mechanisms and ensure HT efficacy.

## Figures and Tables

**Table 1:** Summary of relevant studies of HT in cellular in vitro models.

Study	In Vitro Model	Conditions	HT Health Benefits
Salami et al., 1995 [45]	CuS0_4_-treated LDL samples	10^−5^ M HT	↓ F2-isoprostanes, ↑ Vit E
Visioli et al., 1995 [46]	CuS0_4_-treated LDL samples	HT range 10^−6^–10^−4^ M	↑ Vit E, ↓ lipid peroxidation
Carluccio et al., 2003 [66]	HUVECs and BAECs + LPS, IL-1β, TNFα, or PMA	30 μM HT	↓ VCAM-1, ↓ ICAM-1, ↓ NF-kB, ↓ AP-1, ↓ E-selectin
O’Dowd et al., 2004 [49]	TNF and fMLP-stimulated human neutrophils	10 μM HT	↓ H_2_O_2_
Berrougui et al., 2005 [47]	[^3^H]-Cholesterol-loaded J774 macrophages	HT range 0–25 μM	↑ ABCA1, ↑ apoA-I-mediated cholesterol efflux
Schmitt et al., 2007 [41]	EA.hy926	0.1–100 µM HT for 24 h	No effect on endothelial NO bioavailability and eNOS activity
Carluccio et al., 2007 [69]	Hcy-stimulated HUVECs	HT range 0.1–1 μM	↓ VCAM-1, ↓ ROS, ↓ NF-κB
Dell’Agli et al., 2008 [72]	Human platelets	10 μM HT	↓ cAMP-PDE, ↓ platelet aggregation
Zrelli et al., 2011 [52]	VECs + H_2_O_2_	10, 30, 50 μM HT for 24 h	↓ ROS, ↑ CAT, ↑ FOXO3a, ↑ pAMPK
Richard et al., 2011 [62]	LPS-stimulated RAW264.7	25 μM HT	↓ NO, ↓ PGE₂, ↓ IL-1α, ↓ IL-1β, ↓ IL-6, ↓ IL-12, ↓ TNFα, ↓ CXCL10/IP-10, ↓ CCL2/MCP-1, ↓ iNOS, ↓ MMP-9
Zou et al., 2012 [55]	VECs + H_2_O_2_	50 μM HT	↑ pAkt, ↑ p-p38, ↑ pErK, ↑ Nrf2, ↑ HO-1, ↓ ROS
Scoditti et al., 2012 [76]	HUVEC + PMA	10 μM HT	↓ MMP-9, ↓ COX-2, ↓ PGE2, ↓ NF-kB
Rosignoli et al., 2013 [65]	PMA-activated PBMC	100 μM HT	↓ O_2_^−^, ↓ COX-2, ↓ PGE2
Scoditti et al., 2014 [63]	PMA-activated PBMC and U937	HT range 1–10 μM	↓ MMP-9, ↓ COX-2, ↓ PGE₂, ↓ NF-kB, ↓ PKCβ1, ↓ PKC-α
Takeda et al., 2014 [64]	LPS-stimulated mouse peritoneal macrophages	12.5 μg/mL HT	↓ iNOS, ↓ NO
Storniolo et al., 2014 [43]	HG-stimulated ECV304	10 µM HT for 48 h	↓ ROS, ↑ ET-1, ↑ p-eNOS, ↑ NO
Scoditti et al., 2015 [75]	SGBS cells + TNFα	HT range 0.1–20 μM	↓ pJNK, ↑ adiponectin, ↑ PPARγ
Catalán, et al., 2015 [71]	TNF-α-stimulated HAEC	1, 2, 5, 10 μM HT for 24 h	↓ E-selectin, ↓ P-selectin, ↓ VCAM-1, ↓ ICAM-1, ↓ MCP-1
Ozbek et al., 2015 [51]	H9c2 + O_2_^−^	HT range 0.1-10 µg/mL for 24 h	↓ ROS, ↓ pMAPKAPK-2, ↓ pErk1/2, ↑ Hsp27, ↓ c-CASP3
Zrelli et al., 2015 [57]	VECs	HT range 10-100 μM	↑ PI3K/Akt, ↑ pErK, ↑ Nrf2, ↑ HO-1
Tagliaferro et al., 2015 [53]	Hg induced hemolysis of human RBC	HT range 10-80 μM	↓ ROS, ↑ GSH
Officioso et al., 2016 [54]	Hg induced hemolysis of human RBC	HT range 0.1–5 μM	↓ Eryptosis
Atzeri et al., 2016 [48]	Caco-2 + oxidized cholesterol	HT range 2.5–10 μM	↓ ROS, ↑ GSH, ↓ GPx activity
Calabriso et al., 2018 [50]	PMA-stimulated HUVEC and HMEC-1	HT range 1–30 μM	↓TNFα, ↓IL-1β, ↓VCAM-1, ↓ ICAM-1, ↓ mtROS, ↓ ROS, ↓ MDA, ↓ MnSOD
Manna et al., 2019 [67]	Hcy/TNFα-stimulated EA.hy 926	HT range 0.5–2.5 μM	↓ ICAM-1
Wang et al., 2019 [42]	HG-stimulated HUVECs	25, 50, 100 µM HT-NO for 48 h	↑ p-eNOS, ↑ NO, ↓ ROS, ↑ SIRT-1

↓: decrease, ↑: increase, VCAM-1: vascular cell adhesion molecule- 1, ICAM-1: intercellular adhesion molecule-1, NF-κB: Nuclear Factor kappa-light-chain-enhancer of activated B cells, AP-1: Activator protein 1, ABCA1: ATP-binding cassette transporter A1, NO: nitric oxide, eNOS: endothelial Nitric Oxide Sinthase, ROS: Reactive Oxygen Species, CAT: catalase enzyme, FOXO3a: Forkhead box O3, AMPK: AMP-activated protein chinasi, PGE2: Prostaglandin E2, TNF: Tumor Necrosis Factor, iNOS: inducible Nitric Oxide Sinthase, CXCL10/IP-10: IFN-γ-inducible protein 10, CCL2/MCP1: chemokine (C-C motif) ligand 2, MMP-9: matrix metalloproteinase 9, Akt: serine/threonine-protein kinases, ERK: extracellular signal-regulated kinases, Nrf2: nuclear factor erythroid 2-related factor 2, HO-1: Heme oxygenase 1, COX: Cyclooxygenase, O_2_^−^: superoxide anion, HG: High Glucose, ET-1: endothelin-1, GPx: Glutathione Peroxidase, fMLP: bacterial chemotactic peptide n-formyl-methionine-leucine-phenylalanine, IL: interleukin, mtROS: mitochondrial ROS, MDA: Malondialdehyde, MnSOD: manganese superoxide dismutase, MAPKAPK-2: Mitogen-activated protein kinase-activated protein kinase 2, Hsp27: Heat shock protein 27, c-CASP3: cleaved caspase-3, PI3K: Phosphoinositide 3-kinases, Hcy: Homocysteine, PDE: Cyclic nucleotide phosphodiesterases, JNK: c-Jun N-terminal kinase, PPAR: peroxisome proliferator-activated receptors, SIRT-1: sirtuin 1.

**Table 2:** Summary of relevant studies on HT supplementation in animal models and obtained effects.

Study	In Vivo Model	Conditions	HT Health Benefits
González-Santiago et al., 2006 [84]	Hyperlipemic rabbits, diet induced for 1 month	4 mg/kg HT for 1 month	↓ TC, ↓ TG, ↑ HDL, ↓ atherosclerotic lesion
Fki et al., 2007 [80]	Wistar rats fed cholesterol-rich diet for 16 w	2.5 mg/kg HT + 10 mg/kg OMW	↓ TC, ↓ LDL, ↑ HDL, ↓ TBARS in liver, heart, kidney, and aorta, ↑ CAT, ↑ SOD1
Rietjens et al., 2007 [82]	Lewis rats, excised aorta	HT range 20–100 μM	Protection against induced impairment of NO-mediated aorta relaxation
Jemai et al., 2008 [79]	Wistar rats fed cholesterol-rich diet for 16 w	3 mg/kg/day HT and triacetylated HT	↓ TC, ↓ TG, ↓ LDL, ↓ TBARS in liver, heart, kidney, and aorta, ↑ HDL, ↑ CAT, ↑ SOD1
González-Correa et al., 2008 [85]	Wistar rats	1, 5, 10, 20, 50, 100 mg/kg/day HT, HT-ac or acetylsalicylic acid for 1 w	↓ platelet aggregation, ↓ thromboxane B2, ↓ prostacyclin, ↑ NO
Cao et al., 2014 [81]	C57BL/6J mice high fat diet fed for 17 w	10 and 50 mg/kg/day HT for 17 w	↓ body and organs weight, ↓ HOMA-IR index, ↓ leptin, ↓ IL-6, ↓ CRP, ↓ TG, ↓ HDL, ↓ LDL, ↓ SREBP-1c, ↓ FAS, ↑ SOD1
	db/db metabolic syndrome mice	10 mg/kg/day HT or 225mg/kg/day metformin for 8 w	↓ TG, ↓ TC, ↓ HDL, ↓ LDL, ↓ MDA, ↑ glucose tolerance
Tabernero et al., 2014 [83]	Wistar rats high cholesterol-fed	25 mg/Kg/day HT, HT-ac, HT-et for 8 w	↓ TC, ↓ LDL, ↓ glucose, ↓ insulin, ↓ leptin, ↓ MDA, ↓ TNFα, ↓IL-1β, ↓ MCP-1, ↑ ORAC
Wang et al., 2019 [42]	KM diabetic mice, streptozotocin-induced	77 mg/kg/day HT for 4 w	↓ blood glucose, ↓ MDA, ↑ NO, ↑ SOD1

↓: decrease, ↑: increase, TC: Total cholesterol, TG: Triglycerides, LDL: low density lipoprotein, HDL: high density lipoprotein, w: weeks, CAT: catalase enzyme, OMW: phenolic-rich extract of olive mill wastewaters, SOD1: superoxide dismutase enzyme 1, MDA: Malondialdehyde, NO: nitric oxide, HT-ac: acetate, HT-et: ether, TNFα: Tumor necrosis index, CRP: C reactive protein, SREBP-1c: sterol regulatory element-binding transcription factor 1c, FAS: fatty acid synthase, TBARS: thiobarbituric acid-reactive substances, ORAC: oxygen radical scavenging capacity.

**Table 3:** Summary of relevant studies on HT supplementation in humans and its reported effects.

Study	Population	Conditions	HT Health Benefits
Camargo et al., 2010 [100]	No. 20 metabolic syndrome patients, age range 40–70	0.2 μmol g^−1^ and 45.4 μmol g^−1^ HT, single dose	↓ IL-1β, ↓ IL-6, ↓ PTGS2, ↓NF-κB/MAPK/AP-1 pathway, ↓ chemokines
de Bock et al., 2013 [99]	No. 46 overweight men, age range 35–55	9.7 mg/day HT and 51.1 mg/day oleuropein, for 12 w	↑ insulin sensitivity, ↑ β-cell responsiveness
Colica et al., 2017 [98]	No. 28 healthy subjects, age range 18–65	15 mg/day HT for 3 w	↑ SOD1, ↑ thiol group, ↑ TSA, ↓ MDA, ↓ nitrite, ↓ nitrate, ↓ body fat mass, ↓ suprailiac skinfold, ↓ body weight
Quirós-Fernández et al., 2019 [97]	No. 84 healthy subjects, age range 45–65	9.9 mg/day HT and 195 mg/day punicalagin, for 20 w	↓ ox-LDL, ↓ SYS and DIA blood pressure

↓: decrease, ↑: increase, PTGS2: Prostaglandin-Endoperoxide Synthase 2, MAPK; mitogen-activated protein kinase, w: weeks, SOD1: superoxide dismutase enzyme 1, AP-1: Activator protein 1, TSA: total antioxidant status, MDA: malondialdehyde, LDL: low-density lipoproteins, SYS: Systolic, DIA: Diastolic.
